# Disparity in Resilience: The role of educational attainment and *APOE ε4* in Alzheimer Disease in a Puerto Rican cohort

**DOI:** 10.1002/alz.091642

**Published:** 2025-01-09

**Authors:** Kara L. Hamilton‐Nelson, Farid Rajabli, Dingtian Cai, Larry D. Adams, Pedro R. Mena, Katrina Celis, Jose Javier Sanchez, Glenies S Valladares, Patrice L. Whitehead, Heriberto Acosta, Katalina F McInerney, Rhoda Moise, Anthony J. Griswold, Gary W Beecham, Briseida E. Feliciano‐Astacio, Jeffery M. Vance, Azizi A Seixas, Michael L. Cuccaro, Margaret A Pericak‐Vance

**Affiliations:** ^1^ John P. Hussman Institute for Human Genomics, University of Miami Miller School of Medicine, Miami, FL USA; ^2^ Dr. John T. Macdonald Foundation Department of Human Genetics, University of Miami Miller School of Medicine, Miami, FL USA; ^3^ John P. Hussman Institute for Human Genomics, Miller School of Medicine, Miami, FL USA; ^4^ John P. Hussman Institute for Human Genomics, University of Miami Miller School of Medicine, Miami, FL, USA, Miami, FL USA; ^5^ Carribean Center for the study of memory and cognition, San Juan, PR USA; ^6^ University of Miami Miller School of Medicine, Miami, FL USA; ^7^ Department of Psychiatry and Behavioral Sciences, University of Miami Miller School of Medicine, Miami, FL USA; ^8^ John T. MacDonald Foundation Department of Human Genetics, University of Miami, Miami, FL USA; ^9^ Universidad Central del Caribe, Bayamón, PR USA; ^10^ John P. Hussman Institute for Human Genomics, Miami, FL USA; ^11^ Department of Neurology, University of Miami Miller School of Medicine, Miami, FL USA; ^12^ Department of Informatics and Health Science Data, University of Miami Miller School of Medicine, Miami, FL USA; ^13^ 1501 NW 10th Avenue, Miami, FL USA

## Abstract

**Background:**

Cognitive resilience research in African Americans (AA) shows that higher educational attainment (EA) can mitigate the impact of Alzheimer disease pathology (ADP). However, this mitigating effect is less pronounced in *APOE*ε4 carriers. This finding suggests a disparity in resilience influenced by an interplay of educational and genetic factors. Expanding on this, our study examines Puerto Ricans (PR), a population with distinct social and ancestral background, to assess whether similar patterns of disparity are present. Our aim is to explore the role of education as a modifiable risk factor in Alzheimer disease (AD) and to determine whether the *APOEε4* allele leads to differences in resilience among carriers and non‐carriers.

**Methods:**

We studied 455 PR individuals, focusing on their completed education years, plasma pTau181 levels, and *APOE* genotypes. Using the non‐memory components of the Cognitive Dementia Rating scale, we formulated a composite score CDR‐FUNC to capture functional difficulties. EA was classified into low EA(≤9years) and high EA(>9years). We used plasma pTau181 as proxy for ADP. Those with log10(pTau181) >mean+1SD were considered as advanced pTau181 levels. Participants were also categorized based on their *APOE*ε4 carrier status. The Mann‐WhitneyU test was used to evaluate the association between EA and CDR‐FUNC in individuals with advanced pTau181 levels and ε4 allele.

**Results:**

The study found a correlation between EA and CDR‐FUNC in PRs with high pTau181 levels. Individuals with high EA showed better functional ability than those with low EA(p = 0.014). Additionally, we found that the effect of high EA on functional resilience was stronger in ε4 non‐carriers (p = 0.038) compared to ε4 carriers (p = 0.277).

**Conclusions:**

Our study demonstrates that education is a modifiable risk factor for AD in PRs, contributing to resilience against ADP. Notably, we identified a disparity in the protective effect of EA between carriers and non‐carriers of the *APOE*ε4 allele. Revealing patterns like those observed in AA, our research confirms the combined influence of education, genetics, and functional resilience in AD. The results highlight the importance of education and genetic factors across diverse ethnic and ancestral backgrounds. Our findings are essential for creating targeted interventions to improve health outcomes in PRs and diverse populations affected by AD.

